# Ca^2+^ Channel Re-localization to Plasma-Membrane Microdomains Strengthens Activation of Ca^2+^-Dependent Nuclear Gene Expression

**DOI:** 10.1016/j.celrep.2015.06.018

**Published:** 2015-07-02

**Authors:** Krishna Samanta, Pulak Kar, Gary R. Mirams, Anant B. Parekh

**Affiliations:** 1 Department of Physiology, Anatomy and Genetics, University of Oxford, Parks Road, Oxford OX1 3PT, UK; 2 Department of Computer Science, University of Oxford, Oxford OX1 3QD, UK

## Abstract

In polarized cells or cells with complex geometry, clustering of plasma-membrane (PM) ion channels is an effective mechanism for eliciting spatially restricted signals. However, channel clustering is also seen in cells with relatively simple topology, suggesting it fulfills a more fundamental role in cell biology than simply orchestrating compartmentalized responses. Here, we have compared the ability of store-operated Ca^2+^ release-activated Ca^2+^ (CRAC) channels confined to PM microdomains with a similar number of dispersed CRAC channels to activate transcription factors, which subsequently increase nuclear gene expression. For similar levels of channel activity, we find that channel confinement is considerably more effective in stimulating gene expression. Our results identify a long-range signaling advantage to the tight evolutionary conservation of channel clustering and reveal that CRAC channel aggregation increases the strength, fidelity, and reliability of the general process of excitation-transcription coupling.

## Highlights

• Dispersed Orai1 channels weakly activate c-*fos* and NFAT transcription factors
• Channel re-location to ER-PM junctions robustly activates transcription factors
• Orai1-channel clustering strengthens excitation-transcription coupling

## Graphical Abstract

**Figure:**
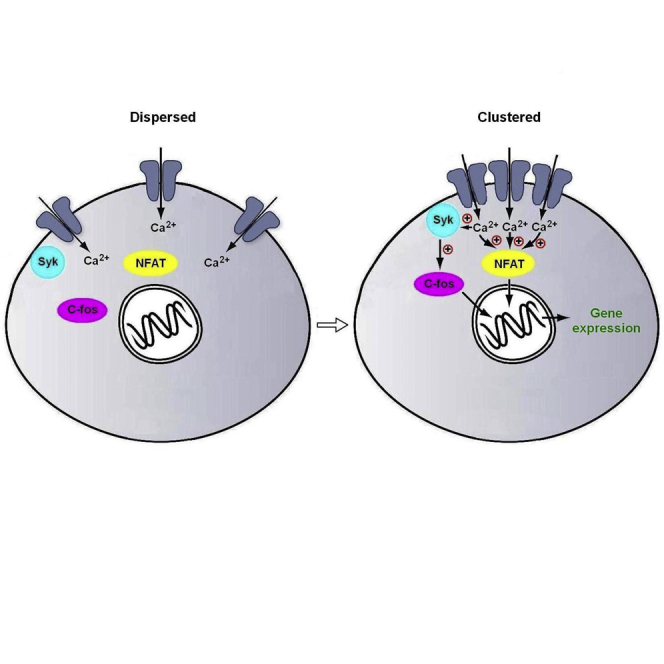

## Teaser

In electrically silent cells, store-operated calcium channels relocate to plasma-membrane microdomains after stimulation. Samanta et al. show that such channel clustering, as opposed to a similar number of dispersed channels, augments the effects of calcium signaling through more robust activation of transcription factors.

## Introduction

Clustering of ion channels is commonly observed in the cell-surface membrane (Hille, 2002). Voltage-dependent Na^+^ channels congregate in the axon hillock where the action potential initiates (Ho et al., 2014), whereas Cav2.2 (N-type) Ca^2+^ channels are concentrated at pre-synaptic sympathetic nerve terminals to drive rapid regulated exocytosis (Khanna et al., 2007). Polarized epithelial cells have an asymmetric distribution of Ca^2+^ channels and Ca^2+^-activated K^+^ and Cl^−^ channels in the basolateral and apical membranes, respectively (Petersen and Tepikin, 2008), forming a “push-pull” mechanism for unidirectional salt transport (Kasai and Augustine, 1990). Ca^2+^-dependent Cl^−^ channels are arranged such that they produce an electrical gradient across the egg that prevents polyspermy. In non-polarized cells, ion channel clustering is maintained, but the signaling advantage conferred by this form of macromolecular crowding is unknown.

One of the most poignant examples of ion channel confinement within a membrane microdomain is that of the store-operated Ca^2+^ release-activated Ca^2+^ (CRAC) channel, which represents a major route of Ca^2+^ entry in eukaryotic cells. The channels open after a fall in free calcium concentration within the ER, as occurs physiologically following stimulation of cell-surface receptors that increase the levels of the second messenger inositol trisphosphate (Parekh and Putney, 2005). Following loss of store Ca^2+^, a highly orchestrated and choreographed sequence of events ensues that is initiated by the dissociation of luminal Ca^2+^ from the canonical EF hand of the ER integral membrane proteins Stromal Interaction Molecule (STIM) 1 and 2 (Liou et al., 2005; Roos et al., 2005; Soboloff et al., 2012). STIM proteins then oligomerize and migrate toward the plasma membrane (PM), a process expedited by a lysine-rich domain on the cytoplasmic C terminus of the protein, which binds to membrane polyphosphoinositides (Hogan et al., 2010). Multimeric STIM complexes then aggregate in regions of peripheral ER, located only ∼10–20 nm from the PM, forming clusters or “puncta” when fluorescently tagged STIM1 is expressed (Wu et al., 2006). At these sites, STIM activates PM Orai1 proteins (Feske et al., 2006), identified through site-directed mutagenesis as the pore-forming subunits of the CRAC channel (Prakriya et al., 2006; Vig et al., 2006; Yeromin et al., 2006). STIM traps and gates open Orai1 channels through binding of its CRAC activation domain or STIM1 Orai1 activation region to intracellular C- and N-terminal sites on the Orai1 channel (McNally et al., 2013; Park et al., 2009; Yuan et al., 2009), which leads to a conformational change at the external entrance to the pore (Gudlur et al., 2014).

Ca^2+^ microdomains near open CRAC channels stimulate gene expression in the RBL mast cell line through recruitment of NFAT (Kar et al., 2011, 2012b) and c-*fos* (Di Capite et al., 2009; Ng et al., 2009) transcription factors. By comparing a CRAC channel mutant that is active in the absence of STIM1 and hence does not aggregate at ER-PM junctions with channels that re-localize to these sites, we have examined whether CRAC channel proximity imparts a signaling advantage to excitation-transcription coupling. We show that for a similar number of active channels and thus for the same global rise in cytoplasmic Ca^2+^, channel localization to ER-PM junctions leads to more robust gene expression. Our findings identify a significant benefit to gene expression through confinement of a Ca^2+^ channel to a PM microdomain.

## Results

### CRAC Channels Activate Both c-*fos* and NFAT Transcription Factors

Ca^2+^ microdomains near open CRAC channels in RBL-1 cells activate two transcription factors: c-*fos*, through enhanced protein expression (Ng et al., 2009), and cytoplasmic NFAT, which can be followed functionally through an NFAT-dependent GFP reporter gene (Kar et al., 2011). To confirm that both responses depended on Orai1, we first used a small interfering RNA (siRNA) knockdown approach to reduce expression of channel protein. Activation of CRAC channels with a maximally effective dose of the sarco-endoplasmic reticular Ca^2+^ATPase (SERCA) pump blocker thapsigargin (2 μM) resulted in a sustained cytoplasmic Ca^2+^ rise, due to Ca^2+^ release from the stores followed by Ca^2+^ influx through CRAC channels (Figure S1A). Knockdown of Orai1, which reduced protein levels by ∼60% (Figures S1C and S1D; Singaravelu et al., 2011), significantly diminished the prolonged phase of the Ca^2+^ signal (Figure S1A), consistent with the reduction in CRAC current in these cells under similar knockdown conditions (Singaravelu et al., 2011). The rate of rise of the cytoplasmic Ca^2+^ signal, seen upon readmission of external Ca^2+^ to cells challenged with thapsigargin in Ca^2+^-free solution for 7 min, was also significantly reduced following Orai1 knockdown (Figure S1B; Singaravelu et al., 2011). qPCR measurements of c-*fos* (Figure S1E) and imaging of NFAT-dependent GFP reporter gene expression (Figures S1F and S1G) following CRAC channel activation revealed that both were significantly reduced after knockdown of Orai1. Further evidence that the CRAC channel activated both c-*fos* and NFAT pathways was provided by studies with the channel blocker Synta66 (Ng et al., 2008), which inhibited both c-*fos* and NFAT-dependent gene expression following activation of the channels with thapsigargin (Figures S1E–S1G).

### CRAC-Channel-Dependent Ca^2+^ Microdomains Activate c-*fos* and NFAT through Distinct Signaling Pathways

**Figure 1:**
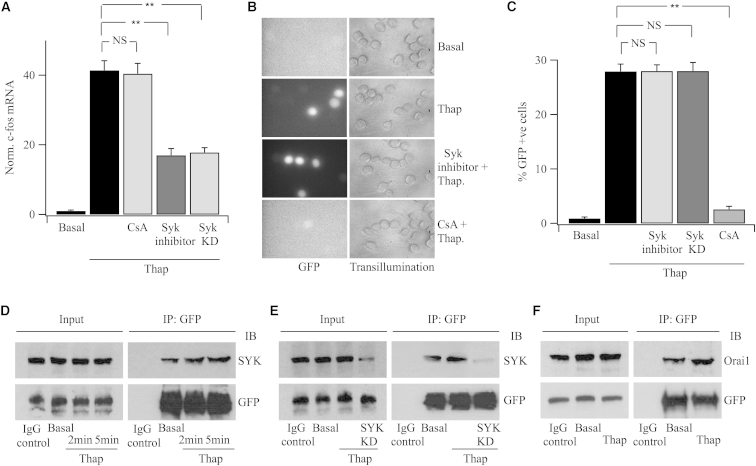
Local Ca^2+^ Entry through CRAC Channels Activates c-*fos* and NFAT through Different Signaling Pathways in RBL-1 Cells (A) Thapsigargin stimulates c-*fos* transcription several-fold above non-stimulated (basal) levels, and this is unaffected by cyclosporine A (1 μM) but significantly reduced by pre-treatment with the Syk inhibitor (10 min; 20 μM) or following knockdown of Syk. C-fos mRNA was measured using qPCR. (B) NFAT-dependent GFP reporter gene expression is unaffected by interfering with Syk but is prevented by cyclosporin A. (C) Aggregate data are compared. Each bar represents data from three independent experiments. ^∗∗^p < 0.01; NS, nonsignificant. (D) Pull-down of Orai1-YFP reveals the presence of Syk under basal conditions, and this increases slightly after stimulation with 2 μM thapsigargin. (E) Knockdown of Syk results in less association with Orai1-YFP. (F) Following pull-down of Syk-YFP, immunoblot reveals the presence of Orai1 under basal conditions, and this increases slightly after store depletion with thapsigargin. In (E) and (F), thapsigargin was present for 5 min before cell lysis. Error bars represent SEM.

NFAT activation requires extensive cytoplasmic dephosphorylation by the protein phosphatase calcineurin, which leads to exposure of a nuclear localization sequence (Hogan et al., 2003). In many cell types, a fraction of calcineurin is bound to AKAP79 at the cell surface. Store depletion leads to association of AKAP79 with Orai1, bringing calcineurin into the realm of the CRAC channel Ca^2+^ microdomain (Kar et al., 2014). Transcription of c-*fos* that occurs in response to local Ca^2+^ entry through CRAC channels requires the non-receptor tyrosine kinase Syk, which then phosphorylates the transcription factor STAT5 (Ng et al., 2009). To see if these pathways overlapped in RBL-1 cells, we interfered with each individually and then examined the impact of this on activation of the other transduction pathway. The calcineurin inhibitor cyclosporine A had no inhibitory effect on c-*fos* expression induced by CRAC channel activity (Figure 1A) but suppressed NFAT-dependent reporter gene expression (Figures 1B and 1C). By contrast, pharmacological inhibition of Syk significantly reduced c-*fos* expression (Figure 1A) but had no effect on the NFAT pathway (Figures 1B and 1C). Judicious use of pharmacological tools therefore suggests local Ca^2+^ entry through CRAC channels activates these transcription factors independently. To strengthen this conclusion, we used an siRNA-based knockdown strategy. Pull-down of recombinant Orai1-yellow fluorescent protein (YFP) revealed an association with Syk in non-stimulated cells (Figure 1D) and this increased further after stimulation with thapsigargin. The association increased slightly with stimulation time (Figure 1D). The interaction was lost after knockdown of Syk (Figure 1E), a maneuver that reduces c-*fos* expression following CRAC channel opening (Figure 1A; Ng et al., 2009). The reverse strategy yielded similar results; pull-down of recombinant Syk-YFP revealed the presence of Orai1 under resting conditions, and this increased after store depletion (Figure 1F). Collectively, these results show that Syk is associated with Orai1 at rest and this interaction increases slightly after store emptying. These data are consistent with our immunocytochemical findings that suggested an association of Syk with the PM, both before and after store depletion (Ng et al., 2009). Knockdown of Syk had no inhibitory effect on the ability of CRAC channels to induce NFAT-dependent reporter gene expression (Figure 1C).

### The V102C Orai1 Mutant Is Constitutively Open and Does Not Form Puncta

Valine 102 is the hydrophobic gate of Orai1 and its mutation to cysteine (V102C) alters gating such that the channel is open under resting conditions, both without the need to deplete stores and in a STIM-independent manner (McNally et al., 2012).

**Figure 2:**
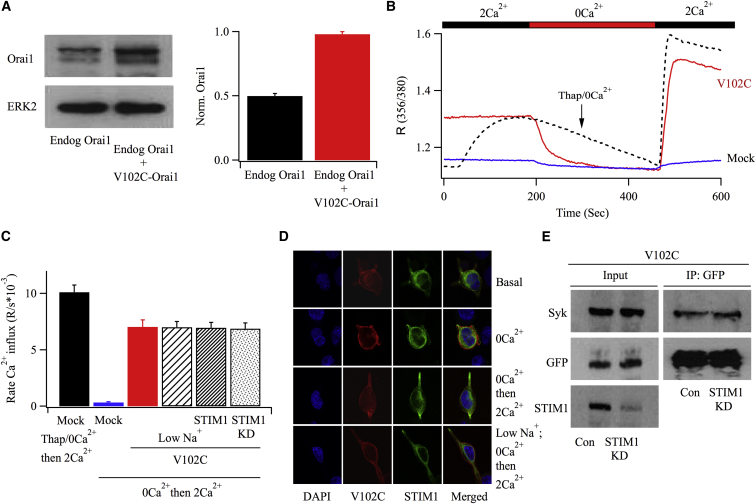
The Mutant V102C-Orai1 Channel Is Constitutively Active and Does Not Form Puncta Characteristic of Store-Operated Orai1 Channels in RBL-1 Cells (A) Western blot compares the amount of endogenous Orai1 protein with levels after expression of V102C-Orai1. The histogram summarizes data from two independent gels. (B) Cytoplasmic Ca^2+^ measurements compare Ca^2+^ entry evoked by V102C channels with that induced by thapsigargin (2 μM). Cells expressing V102C-Orai1 channels were initially maintained in external solution containing 2 mM Ca^2+^ and then perfused with Ca^2+^-free solution for ∼5 min before external Ca^2+^ was readmitted. By contrast, thapsigargin-evoked responses in mock-transfected cells were obtained in Ca^2+^-free solution, and external Ca^2+^ was readmitted ∼7 min later. The mock recording shows a cell exposed simply to Ca^2+^-free solution for 7 min before external Ca^2+^ was readmitted to obtain the basal Ca^2+^ entry rate in the absence of store depletion. (C) Aggregate data for the various conditions are compared. Each bar is the average of between 24 and 38 cells. Low Na^+^ refers to external solution containing 10 mM Na^+^, replaced with Tris^+^. For all bars, cells were exposed to Ca^2+^-free solution for 7 min before external Ca^2+^ was readmitted. (D) Confocal microscopy images compare the distribution of V102C-Orai1-cherry for the conditions shown. STIM1 refers to transfection with STIM1-YFP plasmid. (E) Co-immunoprecipitation studies show that after pull-down of V102C-Orai1-YFP, Syk was detected in the immunoblots, and this association is unaffected by knockdown of STIM1. Error bars represent SEM.

Expression of V102C-Orai1 (untagged) in RBL-1 cells led to an approximate doubling of Orai1 levels (Figure 2A), indicating that the recombinant protein was expressed at a similar level to the endogenous channels. We measured constitutive Ca^2+^ entry following V102C-Orai1 expression by first briefly removing external Ca^2+^ and then measuring the rate of rise of the cytoplasmic Ca^2+^ signal that occurred when external Ca^2+^ was readmitted (Figure 2B). Compared with non-stimulated, mock-transfected cells, where very little Ca^2+^ entry occurred after 5–7 min exposure to Ca^2+^–free solution, prominent Ca^2+^ influx was observed in cells expressing V102C-Orai1 (Figures 2B and 2C). The rate of Ca^2+^ entry for the mutant was slightly (∼30%) but significantly slower than that seen after challenge with a maximally effective concentration of thapsigargin in mock-transfected cells (dotted line in Figure 2B; Figure 2C). Knockdown of STIM1 did not alter the rate of Ca^2+^ influx through V102C-Orai1 channels (Figure 2C), consistent with activity independent of the ER Ca^2+^ sensor. In resting cells, V102C-Orai1-cherry (Figure 2D) was uniformly distributed in the PM with no evidence for the presence of punctate-like fluorescent structures or co-localization with STIM1-YFP (Figure 2D). Neither perfusion in Ca^2+^-free solution for up to 7 min nor subsequent readmission of external Ca^2+^ for 10–20 min altered the distribution of either STIM1 or V102C-Orai1 proteins (Figure 2D). V102C-Orai1 also retained the ability to interact with Syk. Pull-down of V102C-Orai1-YFP with an anti-GFP antibody revealed the presence of Syk in resting cells (Figure 2E), and knockdown of STIM1 did not affect this association (Figure 2E).

### Orai1 Channels Localized to ER-PM Junctions Are More Effective in Stimulating Gene Expression Than Individual V102C-Orai1 Channels

Because V102C-Orai1 channels are active under resting conditions, do not form punctate-like structures in the PM and do not require STIM1, they seem to operate as a series of independent channels. We therefore compared the extent of c-*fos* and NFAT activation induced by Ca^2+^ flux through V102C-Orai1 channels with that through endogenous Orai1 channels localized to ER-PM junctions. For the analysis to be meaningful, it was essential to compare gene expression for similar levels of Ca^2+^ entry. We therefore established the relationship between Ca^2+^ entry and thapsigargin concentration and from this identified a concentration of thapsigargin that generated a rate of Ca^2+^ influx through endogenous Orai1 channels that was identical to that evoked by V102C-Orai1. Having obtained this, we could then compare c-*fos* expression and NFAT activation induced by V102C-Orai1 channels with that evoked by the dose of thapsigargin that gave a similar rate of Ca^2+^ entry but that caused re-localization of endogenous channels to ER-PM junctions.

**Figure 3:**
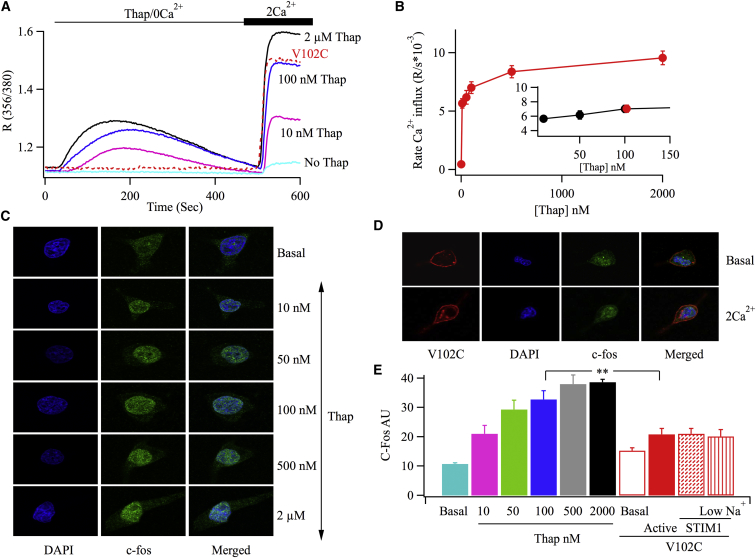
CRAC Channel Re-localization following Store Depletion Is More Effective Than Dispersed V102C-Orai1 Channels in Evoking c-*fos* Gene Expression in RBL-1 Cells (A) Store-operated Ca^2+^ entry is compared following stimulation with different concentrations of thapsigargin. Experiments with V102C-Orai1, carried out with the same preparations of cells, are shown in red. (B) Aggregate data are summarized. Each bar is the average of between 19 and 33 cells. The inset compares the rate of Ca^2+^ entry through V102C-Orai1 with a range of thapsigargin concentrations that evoked similar rates through native Orai1 channels. (C) Expression of c-*fos* is compared for different thapsigargin concentrations. Basal refers to non-stimulated cells. (D) c-*fos* levels in cells expressing V102C-Orai1 are compared. The images labeled “2 Ca^2+^” represent cells first exposed to Ca^2+^-free solution for 7 min and then exposed to 2 mM Ca^2+^ for 7 min, followed by exposure to medium containing La^3+^ for a further 2 hr before fixation. (E) Histogram compares the extent of c-*fos* expression for the conditions shown. Data are the average of between 30 and 53 cells. Error bars represent SEM.

One complication we encountered was that 24–36 hr after transfection with V102C-Orai1 and NFAT-driven GFP reporter gene plasmids, ∼80% of cells were GFP-positive to varying extents. Similarly, c-*fos* levels had increased 24 hr after V102C-Orai1 transfection, although to a level less than that induced by thapsigargin. Constitutive Ca^2+^ entry through the mutant channels, integrated over many hours, is therefore sufficient to stimulate NFAT and c-*fos* gene expression, at least in a sizeable fraction of the cells. This was problematic for two reasons. First, we needed a low background of NFAT/c-*fos* expression in order to compare the relative gene expression capabilities of a defined pulse of Ca^2+^ entry through V102C-Orai1 with that through endogenous Orai1 channels confined to ER-PM junctions. Second, constitutive Ca^2+^ entry through V102C-Orai1 could lead to Ca^2+^-dependent inactivation of the channel, thus diminishing the ability of Orai1 to stimulate gene expression. We therefore adopted the La^3+^ approach that has been used to prevent constitutive Ca^2+^ influx through recombinant Orai1 channels following transfection with the CRAC channel activation domain of STIM1 (Park et al., 2009). Immediately after transfection, RBL-1 cells (transfected with V102C-Orai1 or mock-transfected) were placed in culture medium containing the reversible CRAC channel blocker La^3+^ for the following 24 hr and then loaded with fura-2 in standard Ca^2+^-containing external solution supplemented with La^3+^. Cells were then washed in Ca^2+^- and La^3+^-free external solution containing 0.1 mM EGTA. Application of different concentrations of thapsigargin in Ca^2+^-free solution led to Ca^2+^ release from internal stores, and readmission of external Ca^2+^ 7 min after stimulation resulted in Ca^2+^ influx (Figure 3A). The rate of rise of the Ca^2+^ signal following challenge with 2 μM thapsigargin (Figure 3B) was similar to that seen in control RBL-1 cells not exposed to La^3+^ (Figure S1B), demonstrating that the effects of La^3+^ exposure on Orai1 channels were fully reversible. The relationship between thapsigargin concentration and the rate of Ca^2+^ entry is summarized in Figure 3B. Readmission of external Ca^2+^ to cells exposed to Ca^2+^-free solution for the same period of time but in the absence of thapsigargin (∼7 min) resulted in very little basal Ca^2+^ influx (Figure 3A; labeled no Thap.). The rate of Ca^2+^ influx induced by V102C-Orai1 expression (Figure 3A) was identical to that evoked by 102 nM thapsigargin (inset in Figure 3B; red point denotes V102C-Orai1).

To measure c-*fos* expression, we depleted stores with thapsigargin in Ca^2+^-free solution and then readmitted external Ca^2+^ for 7 min to cells that had been cultured in La^3+^, as described above. After this, we placed cells in medium containing La^3+^ for 2 hr, during which time significant c-*fos* protein expression occurs (Ng et al., 2012). Increasing thapsigargin concentration resulted in an increase in nuclear c-*fos* expression (Figures 3C and 3E).

We then compared the extent of c-*fos* expression in response to a fixed pulse of Ca^2+^ entry through V102C-Orai1-cherry for 7 min with that evoked by 100 nM thapsigargin, a concentration close to the 102 nM that elicited a similar rate of Ca^2+^ influx. C-fos expression was slightly elevated in resting cells expressing V102C-Orai1 (Figure 3D) compared with non-transfected resting cells (both labeled Basal in Figure 3E). Following Ca^2+^ entry through V102C-Orai1 channels, c-*fos* expression (bar labeled Active in Figure 3E) increased only modestly above the basal value. By contrast, stimulation with 100 nM thapsigargin induced c-*fos* expression to a level that was significantly higher than that evoked by active V102C-Orai1 channels (Figure 3E). Hence, V102C-Orai1 induces less c-*fos* expression compared with a dose of thapsigargin that elicits a similar rate of Ca^2+^ entry. Consistent with this, stimulation for 5 min with 100 nM thapsigargin led to a significant increase in STAT5 phosphorylation, whereas Ca^2+^ influx through V102C-Orai1 channels for a similar time period was much less effective (Figure S2).

We repeated these experiments in HEK293 cells, because of their high transfection efficiency, low levels of endogenous Orai1, and the ability to transfect twice with minimal damage to the cells. Western blot analysis indicated that the Orai1-YFP and V102C-Orai1-YFP proteins were expressed to similar extents (Figures S3A and S3B). Confocal microscopy images further revealed that V102C-Orai1-cherry was located mainly at the cell periphery, with a similar spatial distribution to Orai1-YFP (Figures S3C and S3D). As with RBL-1 cells, we constructed a dose-response curve to thapsigargin in HEK cells in order to identify the concentration that evoked Ca^2+^ influx at a rate identical to that induced by V102C-Orai1 channels. Ca^2+^ influx to 101 nM thapsigargin closely matched that evoked by V102C-Orai1 (Figures S4A and S4B). Stimulation of wild-type HEK293 cells with 100 nM thapsigargin led to a significant increase in nuclear c-*fos* and this was abolished by knockdown of endogenous Orai1 (Figures S4C and S4F). Overexpression of STIM1 and Orai1-cherry led to a small further increase in thapsigargin-induced c-*fos* expression compared with wild-type cells (Figure S4F). After knockdown of Orai1, subsequent transfection of STIM1 and Orai1 24 hr later rescued c-*fos* expression to thapsigargin (Figures S4D and S4F). Following knockdown of endogenous Orai1 protein, we expressed V102C-Orai1 channels. Readmission of external Ca^2+^ resulted in a small increase in c-*fos* expression above the basal level (Figure S4E), but this was considerably smaller than that evoked by 100 nM thapsigargin (Figure S4F), in the presence of either 2 mM or 5 mM external Ca^2+^.

### Physiologically Induced CRAC Channel Localization to ER-PM Junctions Increases Signal Strength to the Nucleus

**Figure 4:**
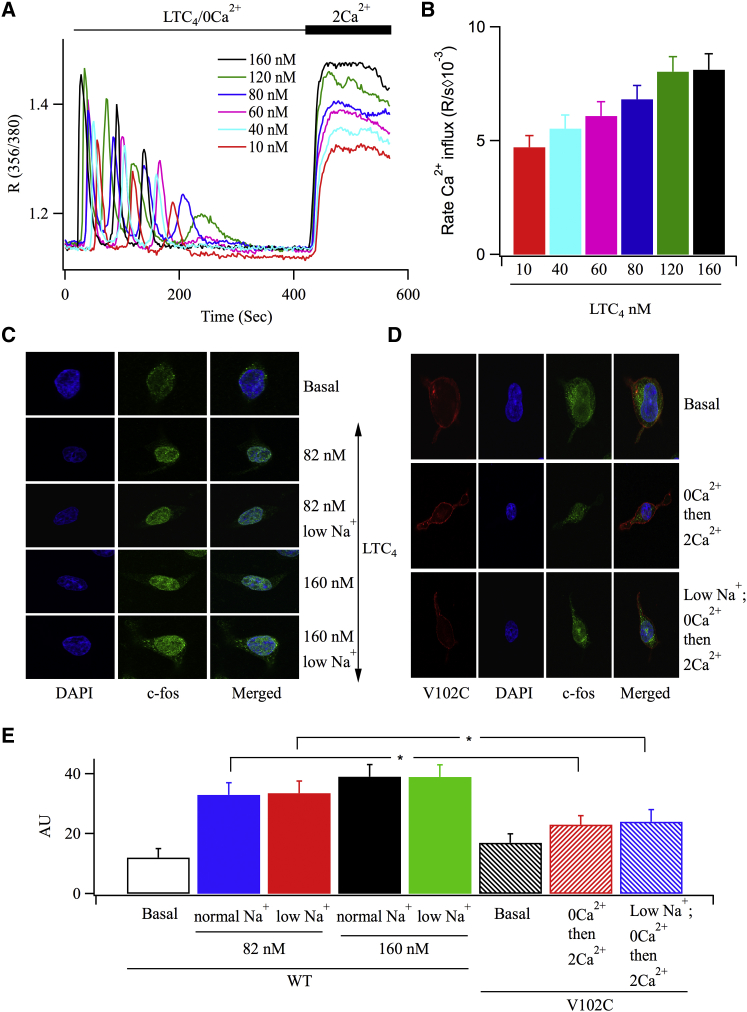
For Similar Ca^2+^ Entry, Physiological Stimulation with LTC_4_ Activates c-*fos* to a Greater Extent Than That Evoked by Constitutive V102C-Orai1 Channels (A) Store-operated Ca^2+^ entry is compared for different concentrations of LTC_4_ (10–160 nM). (B) Aggregate data compare the rate of Ca^2+^ entry for different LTC_4_ concentrations. Each bar is the average of between 25 and 41 cells. We fitted the dose-response curve with a Hill-type equation and found by inspection that an LTC_4_ concentration of 82 nM evoked a rate of Ca^2+^ entry similar to that seen following Ca^2+^ influx through V102C-Orai1 channels. (C) c-*fos* expression is compared for the different conditions shown. Cells were stimulated with the indicated concentration of LTC_4_ in either normal Na^+^-containing (145 mM) or low Na^+^-solution (10 mM). (D) C-fos levels are measured in cells expressing V102C-Orai1 for the conditions shown. (E) Aggregate data from several experiments are compared. All cells used in this figure were cultured in La^3+^-containing medium, as described in the text, to reduce c-*fos* expression following V102C-Orai1 transfection. LTC_4_ was applied to wild-type cells in 2 mM external Ca^2+^ for 20 min in the absence of La^3+^. After 20 min, agonist was removed and La^3+^ reapplied. Cells were then fixed 2 hr later. All data were derived from RBL-1 cells. Error bars represent SEM.

One difficulty with comparing results between V102C-Orai1 and endogenous Orai1 channels stimulated with thapsigargin is that Ca^2+^ clearance by SERCA pumps is impaired under the latter conditions. The local Ca^2+^ rise after channel confinement to ER-PM junctions could therefore be larger and/or have a greater radial spread in the absence of effective Ca^2+^ removal, strengthening activation of the Syk-STAT5 and NFAT pathways. We therefore used a physiological means for activating CRAC channels in the presence of functional SERCA pumps. Stimulation of cysteinyl leukotriene type I receptors with leukotriene C_4_ (LTC_4_) increases inositol trisphosphate levels, resulting in a series of cytoplasmic Ca^2+^ oscillations (Di Capite et al., 2009). As Ca^2+^ is released from the stores, CRAC channels activate and it is the local Ca^2+^ entry through these channels that stimulates c-*fos* expression (Di Capite et al., 2009) and NFAT1 activation (Kar et al., 2011). Knockdown of either STIM1 or Orai1 or pharmacological block of CRAC channels inhibits leukotriene receptor-dependent activation of gene expression (Kar et al., 2012a). Stimulation of RBL-1 cells with LTC_4_ in Ca^2+^-free solution evoked a series of Ca^2+^ oscillations that ran down with time due to the absence of Ca^2+^ influx. Readmission of external Ca^2+^ resulted in a cytoplasmic Ca^2+^ rise as Ca^2+^ entered through the open CRAC channels (Figure 4A). A dose-response curve plotting the rate of Ca^2+^ entry versus LTC_4_ concentration is summarized in Figure 4B. Inspection of this graph identified a LTC_4_ concentration of 82 nM as that which caused a similar rate of Ca^2+^ entry to V102C-Orai1 channels. 82 nM LTC_4_ caused significant c-*fos* expression, both in standard external solution (145 mM Na^+^ and 2 mM Ca^2+^) and in low Na^+^-containing solution (Figure 4C; aggregate data are shown in Figure 4E). The extent of c-*fos* expression following stimulation with 160 nM LTC_4_, a maximally effective dose for c-*fos* induction, was not significantly different from that elicited by 82 nM LTC_4_ (Figures 4C and 4E). By contrast, Ca^2+^ flux through V102C-Orai1 channels evoked considerably less c-*fos* expression, when compared with 82 nM LTC_4_ (Figures 4D and 4E), in either high or low Na^+^-containing solution.

### Localization of V102C-Orai1 Channels to ER-PM Junctions Is More Effective in Activating NFAT and c-*fos* Than Dispersed Channels in the Same Cells

**Figure 5:**
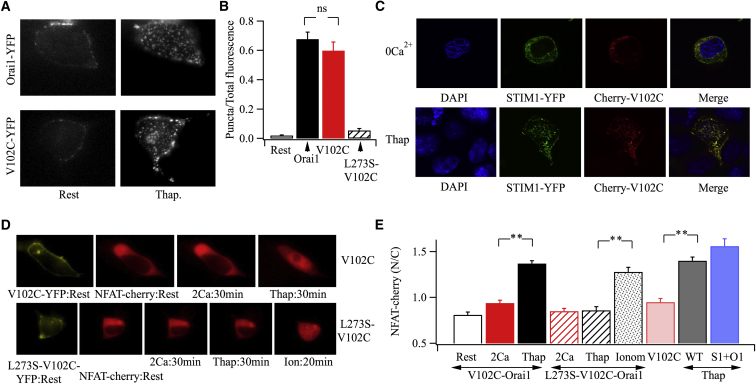
Re-localization of V102C-Orai1 Channels Is More Effective in Activating NFAT Than a Similar Number of Dispersed Channels (A) TIRF images show the distribution of Orai1-YFP or V102C-Orai1-YFP at rest and then after exposure to thapsigargin in Ca^2+^-free solution for 7 min. Endogenous Orai1 had been knocked down prior to transfection with the YFP constructs and untagged STIM1. (B) The histogram compares the ratio of YFP fluorescence in the TIRF field after exposure to thapsigargin divided by the total YFP fluorescence within the same field (basal plus stimulated). (C) Confocal microscopy images compare the distribution of co-expressed STIM1-YFP and V102C-Orai1-cherry at rest and after stimulation with 100 nM thapsigargin in Ca^2+^-free solution for 7 min. (D) NFAT1-cherry migration into the nucleus is compared between a cell expressing V102C-Orai1-YFP with one expressing L273S-V102C-Orai1-YFP. Images labeled “Rest” refer to the distribution of tagged protein prior to stimulation in Ca^2+^-free solution. 2 mM Ca^2+^ was readmitted for 30 min, then 100 nM thapsigargin was applied for 30 min. For the L273S-V102C-Orai1-YFP experiment, 2 μM ionomycin (Ion) was applied after thapsigargin. (E) Aggregate data for the different conditions indicated are compared. The column labeled V102C refers to NFAT1-cherry movement following Ca^2+^ flux through V102C-Orai1 for 60 min. All cells used in the figure are HEK cells. Error bars represent SEM.

One limitation with our approach is that we are comparing the signaling ability of recombinant dispersed V102C-Orai1 channels with endogenous Orai1 channels confined to ER-PM junctions. Although both channels have similar rates of Ca^2+^ entry, the number of functional channels could nevertheless differ or the endogenous channels may have better access to downstream pathways than the V102C-Orai1 channels. To circumvent these concerns, we designed experiments to compare the ability of V012C-Orai1 channels to activate NFAT, first in dispersed mode and then after re-localization to ER-PM junctions in the same cells. To assess V102C-Orai1 distribution, we co-expressed V102C-Orai1-YFP and untagged STIM1 in HEK cells in which Orai1 had been knocked down 24 hr earlier (which abolishes thapsigargin-evoked gene expression; Figure S4C) and used total internal reflection fluorescence (TIRF) microscopy to measure the extent of channel puncta formation. Under resting conditions, V102C-Orai1-YFP channels were dispersed throughout the evanescent field, with no visible puncta (Figure 5A, left-hand panel). Application of 100 nM thapsigargin now led to striking redistribution of the channels into numerous puncta (Figure 5A, right-hand panel), and the increase was similar to that seen when cells expressing Orai1-YFP and untagged STIM1 were stimulated instead (Figure 5A). Analysis of puncta formation, based on the approach described by McNally et al. (2013), is summarized in Figure 5B. Puncta formation after stimulation with 100 nM thapsigargin was not significantly different between V102C-Orai1-YFP and Orai1-YFP channels. These results were confirmed using confocal microscopy, albeit in fixed cells, following expression of V102C-Orai1-cherry with STIM1-YFP (Figure 5C). In resting cells, V102C-Orai1-cherry was distributed across the PM with no evidence for either punctate-like structures or co-localization with STIM1-YFP. After a 5-min treatment with thapsigargin, STIM1 and Orai1 puncta formed and co-localized well (Figure 5C). To measure NFAT activation, we first knocked down endogenous Orai1 channels and then expressed V102C-Orai1-YFP, untagged STIM1, and NFAT1-cherry 24 hr later. Following perfusion with Ca^2+^-free solution for 7 min, Ca^2+^ readmission for 30 min resulted in little NFAT1-cherry migration to the nucleus (Figures 5D and 5E). However, application of thapsigargin now resulted in strong nuclear accumulation of NFAT1-cherry in the same cells (Figures 5D and 5E). The increase in NFAT1-cherry movement was neither a time-dependent phenomenon nor a consequence of continuous Ca^2+^ influx through V102C-Orai1 channels over 60 min, because Ca^2+^ entry through the channels but in the absence of thapsigargin failed to cause NFAT movement (Figure 5E; bar labeled V102C). Collectively, these results are consistent with the idea that localization of V102C-Orai1 to ER-PM junctions is considerably more effective in activating NFAT than dispersed channels in the same cells. NFAT-cherry migration in response to 100 nM thapsigargin in cells with endogenous Orai1 and STIM1 levels or after their overexpression is included in Figure 5E.

**Figure 6:**
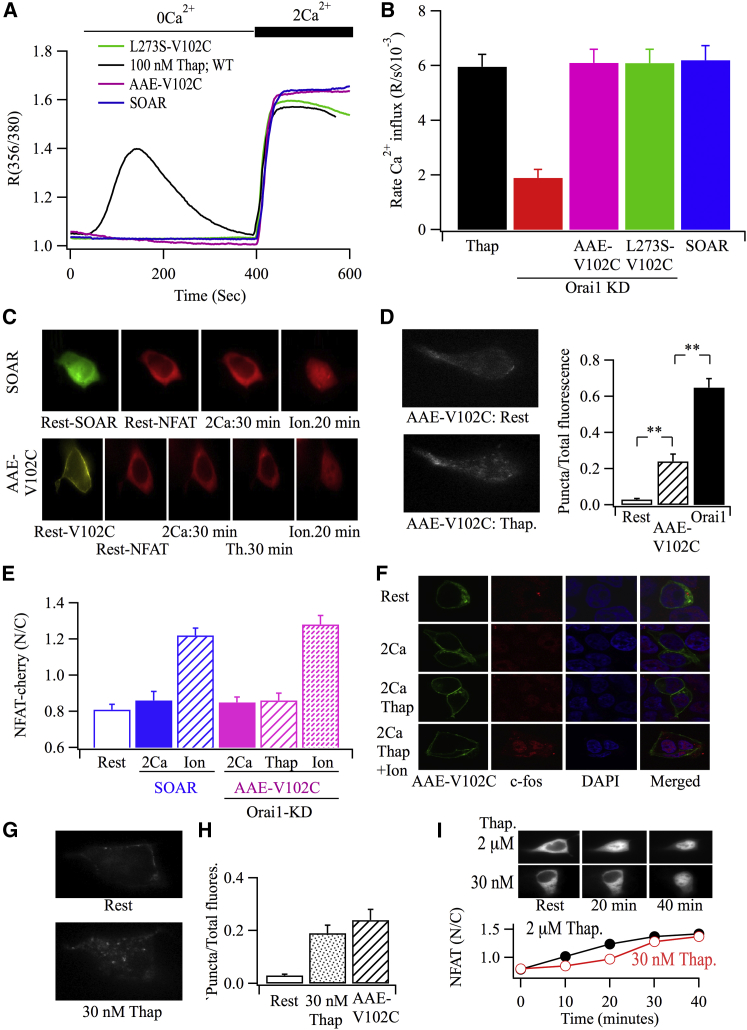
STIM1 Gating Enhances Nuclear Signaling (A) Ca^2+^ influx for the various conditions indicated are compared in HEK cells. Ca^2+^ influx to 100 nM thapsigargin or expression of the SOAR domain was measured through endogenous Orai1 channels. (B) Aggregate data from several experiments are compared. Each bar is the average of between 24 and 39 cells. (C) NFAT1-cherry movement is shown in cells co-transfected with either SOAR-GFP or AAE-V102C-Orai1-YFP (representing ^81^AARAE^85^-V102C-Orai1-YFP) and untagged STIM1. (D) TIRF images are shown for a cell expressing AAE-V102C-Orai1-YFP and untagged STIM1 at rest and then after 7 min treatment with 100 nM thapsigargin. The histogram summarizes data from 17 cells (AAE-V102C-Orai1-YFP) and 12 cells (Orai1-YFP), respectively. Rest values for AAE-V102C-Orai1 and Orai1 were similar and have been pooled together. (E) Aggregate data are compared for the conditions shown. Orai1 was knocked down 24 hr before transfection with AAE-V102C-Orai1 and NFAT1-cherry. 2 Ca^2+^ was applied for 30 min, as was thapsigargin (100 nM) and ionomycin (2 μM). Each bar represents data from between 11 and 23 cells. Rest levels for SOAR and AAE-V102C were not significantly different and have been combined. (F) c-*fos* protein expression is compared for the different conditions in cells expressing AAE-V102C-Orai1-YFP. (G) TIRF images for a cell expressing Orai1-YFP and untagged STIM1 are shown at rest and after stimulation with 30 nM thapsigargin. (H) Aggregate data are compared. AAE-V102C-Orai1-YFP data are taken from Figure 6D. The 30 nM thapsigargin bar reflects nine cells. (I) NFAT1-cherry nuclear movement in response to 30 nM and 2 μM thapsigargin are compared. Each bar denotes data from between 9 and 12 cells. All cells in this figure are HEK cells. Error bars represent SEM.

Confinement of V102C-Orai1 channels to ER-PM junctions after store depletion is driven by accumulation of STIM1 at these sites. The CRAC-activating domain or STIM1 Orai1 activating region (SOAR) of STIM1 interacts with a leucine-rich coiled-coil motif on the C terminus of Orai1. Mutation of leucine 273 (L273S) or leucine 276 (L276D) in this region prevents STIM-Orai interaction as well as CRAC channel activation (McNally et al., 2013; Muik et al., 2011). To test whether the activation of NFAT following store depletion in cells expressing V102C-Orai1 channels was indeed due to channel localization in ER-PM junctions, we expressed V102C-Orai1 channels in which L273 had been mutated (L273S-Orai1) together with STIM1 and NFAT1-cherry in HEK cells in which Orai1 had been knocked down. Constitutive Ca^2+^ influx through the channels was similar to that seen with V102C-Orai1 (compare Figure 6A with Figure S4B) and NFAT migration was undetectable (Figures 5D and 5E). Consistent with previous reports, TIRF microscopy revealed the absence of L273S-V102C-Orai1-YFP puncta after store depletion (Figure 5B). Accumulation of NFAT1-cherry within the nucleus following stimulation with thapsigargin was also suppressed when L273S-V102C-Orai1-YFP was expressed (Figures 5D and 5E). However, subsequent exposure to a concentration of ionomycin that raises cytoplasmic Ca^2+^ to high levels independent of CRAC channels rescued NFAT1-cherry migration in thapsigargin-treated cells (Figures 5D and 5E).

Similar results were seen when c-*fos* expression was measured (Figure S5). Dispersed V102C-Orai1 channels were ineffective, whereas re-localization to ER-PM junctions in response to 100 nM thapsigargin stimulation led to strong expression of c-*fos* (Figure S5C). Induction of c-*fos* to 100 nM thapsigargin was prevented when the L273S-V102C-Orai1 construct was expressed instead (Figures S5A and S5C).

### Orai1 Activation by the SOAR/CAD STIM1 Fragment Does Not Stimulate NFAT Migration

One explanation for the preceding data is that localization to ER-PM junctions renders V102C-Orai1 channels more potent in signaling than when they are dispersed. However, alternative explanations are possible. STIM1-dependent gating might affect the association of dispersed V102C-Orai1 channels with downstream proteins and thus increase the efficiency of downstream signaling. To test this, we expressed the SOAR domain of STIM1, which binds to and activates Orai1 channels in the absence of store depletion and therefore without channel re-localization to ER-PM junctions (Yuan et al., 2009). SOAR-GFP expressed mainly near the cell periphery, as previous reported (Yuan et al., 2009). Constitutive Ca^2+^ entry through endogenous Orai1 channels following expression of the SOAR domain was prominent (Figures 6A and 6B) and occurred at a rate similar to that of ∼100 nM thapsigargin (Figure S4). However, endogenous Orai1 channels failed to stimulate NFAT1-cherry migration to the nucleus when activated by the SOAR domain (Figures 6C and 6E). NFAT movement could be activated subsequently by ionomycin (Figures 6C and 6E). Expression of the SOAR domain also failed to induce c-*fos* expression (Figures S5B and S5C). Hence, STIM1 binding per se to dispersed Orai1 channels is not sufficient for downstream nuclear signaling.

### Tuning of Local Ca^2+^ by STIM1

The high Ca^2+^ selectivity of Orai1 channels is reduced in the V102C-Orai1 mutant, with the fractional Ca^2+^ current in the latter being ∼40% of wild-type channels (McNally et al., 2012). V102C-Orai1 channels exhibit a significant conductivity to Na^+^ (McNally et al., 2012). We therefore considered that V102C-Orai1 channels were more effective in activating NFAT after store depletion by virtue of the increase in Ca^2+^ selectivity that occurs following STIM1 association, which would lead to a stronger local Ca^2+^ signal. Several arguments suggest that this cannot wholly explain our results. First, tethering of the CRAC activation domain to Orai1 V102C rescued the high Ca^2+^ selectivity (McNally et al., 2013), but the closely related SOAR domain was ineffectual in activating NFAT1 (Figures 6C and 6E). Second, lowering external Na^+^ to 10 mM did not change the rate of Ca^2+^ entry through V102C-Orai1 channels (Figure 2C), the distribution of V102C-Orai1 channels (Figure 2D), or c-*fos* expression (Figure 3E). Third, raising external Ca^2+^ to 5 mM while lowering Na^+^ to 10 mM did not increase c-*fos* in cells expressing dispersed V102C-Orai1 channels (Figures S4E and S4F).

STIM1 also binds to the N terminus of Orai1 adjacent to transmembrane domain 1, and this leads to channel gating. A short stretch of amino acids (^81^LSRAK^85^) is essential for the gating step (Gudlur et al., 2014). A single point mutation within this stretch (K85A or K85E) reduces FRET signals between STIM1 and Orai1 by ∼30%–50%, reduces Orai1 puncta formation by ∼70% and abolishes channel activation (Lis et al., 2010; McNally et al., 2013). This raises an interesting question: is Orai1-channel re-localization sufficient for downstream signaling, or is the increase in Ca^2+^ selectivity induced by full-length STIM1 binding to the N terminus additionally required? The fact that some Orai1-YFP puncta form after store depletion despite mutations within the ^81^LSRAK^85^-Orai1 stretch afforded an opportunity to distinguish between these possibilities. We expressed a V102C-Orai1 construct in which key residues within the gating stretch had been mutated (^81^AARAE^85^-V102C-Orai1) (Gudlur et al., 2014) and examined whether this impacted on NFAT and c-*fos* activation, as the mutated channel remains constitutively active. We first measured puncta formation using TIRF microscopy. Hardly any puncta were seen in resting cells expressing ^81^AARAE^85^-V102C-Orai1-YFP, but these became more apparent after store depletion (Figure 6D). Although ^81^AARAE^85^-V102C-Orai1-YFP formed puncta, these were fewer or weaker than those seen with V102C-Orai1-YFP or Orai1-YFP in response to the same stimulus intensity (Figure 5B versus Figure 6D). Following application of 100 nM thapsigargin to cells expressing ^81^AARAE^85^-V102C-Orai1-YFP and untagged STIM1 and NFAT1-cherry and in which Orai1 had been knocked down 24 hr earlier, no detectable nuclear accumulation of NFAT occurred (Figures 6C and 6E). Similar results were obtained when c-*fos* expression was measured instead (Figure 6F; aggregate data summarized in Figure S5C). These results support the concept that re-localization of V102C-Orai1 channels to ER-PM junctions channels alone might not be sufficient for nuclear signaling. To probe this further, we sought a concentration of thapsigargin that induced an increase in Orai1-YFP puncta in the TIRF field similar to that seen with ^81^AARAE^85^-V102C-Orai1-YFP. 30 nM thapsigargin induced an increase in Orai1-YFP fluorescence similar to that induced by 100 nM thapsigargin in cell expressing ^81^AARAE^85^-V102C-Orai1-YFP (Figures 6G and 6H). However, 30 nM thapsigargin triggered clear movement of NFAT1-cherry into the nucleus (Figure 6I). Although prominent, this NFAT movement was nevertheless slower initially than that seen with 2 μM thapsigargin (Figure 6I).

### Endogenous ER-PM Junctions in RBL Cells Are Estimated to Contain Approximately Five CRAC Channels

**Figure 7:**
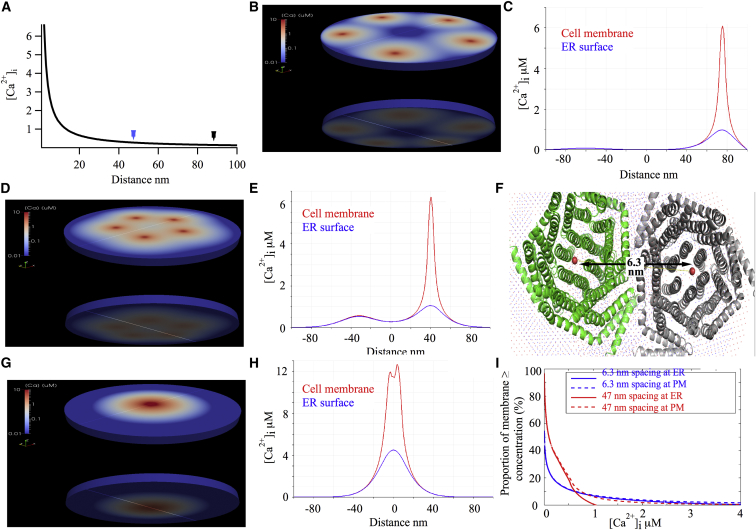
Simulations of Ca^2+^ Concentration within the ER-PM Junction (A) Spatial profile of local Ca^2+^ near a single open CRAC channel. The x axis refers to distance from the channel mouth. (B) Color-coded depiction of [Ca^2+^] in an ER-PM tubule when channels are spaced 88 nm apart. Upper image reflects Ca^2+^ at the cytosolic face of the PM. Lower image is Ca^2+^ at the ER surface. (C) Graphical simulation of local Ca^2+^ for the condition in (B). (D and E) As in (B) and (C), but now with channels 47 nm apart. (F) Superposition of two Orai1-channel pores, based on the crystal structure. (G and H) As in panel (B) and (C), but now with an inter-channel distance of 6.3 nm. (I) The graph depicts the fraction of ER or cell-surface membrane that is exposed to a particular Ca^2+^ value (x axis).

From overexpression studies, it has been estimated that individual puncta contain ∼1,300 CRAC channels (Ji et al., 2008). However, the number of native channels that gather at ER-PM junctions remains unknown. In an effort to obtain a rough estimate, we analyzed our previous electron micrographs that revealed ER-PM junctions through identification of recombinant STIM1 (Singaravelu et al., 2011). The length of these tubules is between ∼50 and 200 nm, and they occupy ∼4% of the cell periphery. These values are in good agreement with the original findings from T cells, where an average length of 150 nm was reported (Wu et al., 2006). For an RBL-1 cell with a membrane capacitance of typically 12 pF and considering a tubule as a spot with a diameter of 200 nm, we estimate ∼1,600 ER-PM junctions per cell. With the RBL-1 cell having a macroscopic CRAC current of −50 pA at −80 mV, a unitary current of ∼−2 fA (Chang et al., 2008; Zweifach and Lewis, 1993), correcting for Ca^2+^ flux in physiological solution (2 mM external Ca^2+^) and taking an open probability of 0.8 (Prakriya and Lewis, 2006), we calculate ∼7,500 endogenous functional channels per cell. If the channels are homogenously distributed throughout the PM, the typical distance between CRAC channels will be ∼470 nm. Assuming all ER-PM junctions are occupied and contain a similar number of channels, then after store depletion, one ER-PM junction in an RBL-1 cell will typically have between four and five CRAC channels. The average inter-channel distance within a junction, assuming no physical coupling between any two channels picked at random, would fall to ∼88 nm. The mean distance between any one channel picked at random and its nearest neighbor in a junction is ∼47 nm. To develop these concepts more formally, we calculated local Ca^2+^ near the mouth of an open CRAC channel as well as the Ca^2+^ concentration 15 nm away, corresponding to the ER surface. The spatial profile of local Ca^2+^ near one open CRAC channel is shown in Figure 7A. We first placed five channels within a single ER-PM junction, with each one 88 nm from its nearest neighbor (Figure 7B; upper profile denotes Ca^2+^ below the PM, lower panel shows the profile at the face of the ER). The simulated Ca^2+^ concentration for a line scan across the center of the junction is shown in Figure 7C. The center of the junction serves as the frame of reference. For much of the junction, local Ca^2+^ both below the PM and at the apposed ER surface is low, as few channels are close by. However, local Ca^2+^ at both surfaces increases steeply close to a single channel that happens to be near the central line. The Ca^2+^ profile here is similar to that predicted for a single channel (Figure 7A), indicating little overlap of Ca^2+^ microdomains. The pattern changes slightly when five channels within a junction are placed 47 nm apart (Figure 7D), mimicking the nearest distance between two non-coupled channels at a junction. Now, the bulk level is elevated in the mid-range of the junction and two peaks in Ca^2+^ arise (Figure 7E): a large one, again corresponding to a single channel, and a second, smaller one that represents spillover from a couple of proximal channels. If CRAC channels co-localize at a junction, we estimate the distance between two channel pores to be 63.4 A (∼6.3 nm), based on a linear measure from juxtaposition of the crystal structures of the channel (Hou et al., 2012) (Figure 7F). We therefore positioned five channels at a spacing of 6.3 nm (Figure 7G). The Ca^2+^ profile in the junction changed considerably (Figure 7H). Ca^2+^ immediately below the PM increased to ∼13 μM, and Ca^2+^ at the ER surface rose to >4 μM. Interestingly, the lateral expanse of local Ca^2+^ >10 μM at the face of the PM extended for ∼20 nm from the channel cluster and was >2 μM (a value higher than the bulk Ca^2+^ typically measured in non-excitable cells after maximum stimulation) for ∼40 nm. Re-localization of the low conductance CRAC channels to ER-PM junctions therefore enables a local Ca^2+^ signal to extend several tens of nanometers across the junction. The fraction of PM or outer ER surface membrane that experiences different local Ca^2+^ concentrations is plotted in Figure 7I for different levels of channel spacing. At a spacing of 6.3 nm, almost 10% of the ER surface is exposed to a local Ca^2+^ concentration >1 μM. By contrast, almost all the ER in a junction experiences Ca^2+^ < 1 μM at 43- or 88-nm spacing. The profiles suggest that clustering enables Ca^2+^ sensors or detectors on the apposite ER surface to be exposed to Ca^2+^ levels several-fold more than the bulk level.

## Discussion

Clustering of ion channels is often observed in large cells like neurons to enhance the speed of response and attenuate signal dilution/decay. In non-polarized cells or smaller cells with less complex geometry, channel clustering occurs in a regulated manner, but whether this confers a signaling advantage is unknown. In this study, we show that CRAC channels, which re-localize to regions of PM juxtaposed against the ER following stimulation of the phospholipase C pathway, are more effective in activating c-*fos* gene expression and the NFAT pathway than a similar number of independent CRAC channels distributed more diffusely. The increased signaling strength of CRAC channels confined to ER-PM junctions was not a consequence of greater accessibility to downstream signals such as Syk, the tyrosine kinase that activates c-*fos* through phosphorylation of STAT5, because resting Orai1 channels, activated Orai1 channels, and the constitutive V102C-Orai1 channel mutant all co-immunoprecipitated with the kinase. Furthermore, STIM1 binding to dispersed V102C-Orai1 channels, as evinced by the SOAR domain, still failed to activate c-*fos* or NFAT. The increased efficiency imparted by CRAC channel re-localization likely arises from enhanced local Ca^2+^ signals in the vicinity of each ER-PM junction, raising the amplitude and extending the radial spread of the Ca^2+^ microdomain. Such an increase in size and breadth of the local Ca^2+^ signal would be expected to increase the strength of Syk-STAT5 and calcineurin activation, increasing signal transduction to the nucleus via c-*fos* and NFAT, respectively. Our estimates of Ca^2+^ concentration in ER-PM junctions suggest that CRAC channel re-localization can increase local Ca^2+^ to levels at least an order of magnitude greater than bulk Ca^2+^. Corralling low-conductance Ca^2+^ channels into PM microdomains thus increases signaling strength by significantly enhancing the local Ca^2+^ concentration.

Although our results reveal that re-localization of CRAC channels to ER-PM junctions is important, they do not address the question of whether the channels need to cluster together tightly. The analysis in Figure 7 suggests that dispersed channels within an ER-PM junction cover a greater area with modestly elevated calcium in the range up to several hundred nM yet still provide several μM Ca^2+^ to some parts of the cytosolic face of the PM. It is therefore interesting to consider that varying recruitment of Orai1 channels to a cluster might be an effective way to engender varying patterns of sub-plasmalemmal Ca^2+^ at a junction. Co-localization of all five channels will result in very high but local Ca^2+^, whereas varying combinations of tightly clustered with dispersed channels will yield different spatial Ca^2+^ signatures. The extent of Orai1 recruitment to a tight cluster might afford a means to activate different Ca^2+^ sensors within a junction.

Our results, derived from experiments with a low concentration of thapsigargin, suggest that channel re-localization, although necessary, is not sufficient to strengthen CRAC channel signaling to the nucleus. 30 nM thapsigargin induced a similar increase in Orai1-YFP fluorescence within the TIRF field as ^81^AARAE^85^ -V102C-Orai1-YFP, but the lower thapsigargin concentration was significantly more effective in activating NFAT. Although STIM1 associates with the C terminus of ^81^AARAE^85^-V102C-Orai1, mutations within the stretch between amino acids 81 and 85 weaken the interaction at the N terminus. It is therefore possible that this reduced interaction at the gating hinge impairs the increase in Ca^2+^ selectivity of Orai1 that occurs upon STIM1 gating, rendering ^81^AARAE^85^-V102C-Orai1 less efficient in generating or delivering the local Ca^2+^ signal. In this regard, it is tempting to speculate that STIM1 is a master regulator of Orai1, not only re-localizing and gating the protein, but also increasing the Ca^2+^ selectivity of Orai1 (McNally et al., 2012), which further serves to enhance the impact of channel re-localization on downstream signaling pathways. However, we submit that the lifetime of STIM1-Orai1 puncta may be longer than that of STIM1-^81^AARAE^85^-V102C-Orai1, which could impact downstream signaling independent of a change in Ca^2+^ selectivity of the channels. We did not observe more rapid disaggregation of ^81^AARAE^85^-V102C-Orai1-YFP puncta compared with those formed by Orai1-YFP, although it is possible that the former flickered more and therefore were missed with our acquisition rate of 0.25–0.5 Hz. A further possibility is that the ^81^AARAE^85^-V102C-Orai1 is less able to interact with downstream signaling molecules, despite forming puncta similar in extent to those induced by 30 nM thapsigargin.

Our findings reveal a significant functional advantage to re-localization of CRAC channels to ER-PM junctions, enabling robust signaling to spatially distant targets.

## Experimental Procedures

### Cell Culture and Transfection

Rat basophilic leukemia (RBL-1) and HEK293 cells were bought from the ATCC and cultured (37°C, 5% CO_2_) in DMEM with 10% fetal bovine serum and 2 mM L-glutamine and penicillin-streptomycin, as previously described (Kar et al., 2012a). RBL-1 cells were transfected using the AMAXA system, and HEK293 cells were transfected using the Lipofectamine method, as described previously (Kar et al., 2011).

### Ca^2+^ Imaging

Ca^2+^ imaging experiments were carried out at room temperature, using the IMAGO CCD camera-based system from TILL Photonics (Di Capite et al., 2009). Cells were loaded with Fura 2-AM (2 μM) for 40 min at room temperature in the dark and then washed three times in standard external solution of 145 mM NaCl, 2.8 mM KCl, 2 mM CaCl_2_, 2 mM MgCl_2_, 10 mM D-glucose, and 10 mM HEPES (pH 7.4) with NaOH. Ca^2+^-free solution contained 145 mM NaCl, 2.8 mM KCl, 2 mM MgCl_2_, 10 mM D-glucose, 10 mM HEPES, and 0.1 mM EGTA (pH 7.4) with NaOH. For the low Na^+^ external solution, NaCl was reduced to 10 mM and replaced with 135 mM Tris base. Cells were alternately excited at 356 and 380 nm (20-ms exposures), and images were acquired every 2 s. Ca^2+^ signals are plotted as R, which denotes the 356/380 nm ratio. Further details are provided in Supplemental Experimental Procedures.

### TIRF Microscopy

HEK293 cells expressing Orai1-YFP, V102C-Orai1-YFP, or mutants thereof were illuminated with 488-nm laser light. Light reflected from the back focal plane was detected with a ×100 oil-immersion objective, and images were captured with 1 × 1 pixel binning. YFP fluorescence was measured before and then after stimulation with thapsigargin (concentrations indicated in text) for each cell. YFP fluorescence was measured in Image J along three lines drawn across each cell, as described in Supplemental Experimental Procedures.

### Nuclear NFAT1-GFP

NFAT1-GFP levels in the cytosol and nucleus were measured using the IMAGO charge-coupled device camera-based system from TILL Photonics, with a ×100 oil-immersion objective (Kar et al., 2012a). Regions of interest of identical size were drawn in the cytosol and nucleus of each cell, and the nuclear/cytosolic ratio of NFAT-GFP was calculated. Only one to three cells per field of view on each coverslip were used, and translocation was measured in these cells for up to 90 min.

To prevent constitutive Ca^2+^ influx through V102C-Orai1 and associated mutants from stimulating gene expression during the culture period after transfection, we used the La^3+^ method (Park et al., 2009), where the CRAC channel blocker La^3+^ was added to the culture medium and then maintained until shortly before the onset of experiments. Control cells as well as those expressing Orai1-YFP, which served as appropriate controls, were also exposed to La^3+^ under identical conditions.

### Gene Reporter Assay

24–36 hr after transfection with the EGFP-based reporter plasmid that contained an NFAT promoter (a gift from Dr. Yuri Usachev, University of Iowa), cells were stimulated with thapsigargin and the percentage of cells expressing EGFP measured subsequently (∼24 hr later), as described in Supplemental Experimental Procedures.

### siRNA Knockdown

siRNAs against Orai1 and STIM1 were from Origene and siRNA against SYK was from Invitrogen, as reported previously (Ng et al., 2009).

### Confocal Microscopy

After treatment, cells were fixed in 4% paraformaldehyde at room temperature and permeabilized with PBS/Triton 0.5%. Cells were stained overnight at 4°C with c-*fos* primary antibody (Santa Cruz Biotechnology), as described in Supplemental Experimental Procedures. Nuclei were counterstained with DAPI.

### Co-immunoprecipitation and Western Blotting

48 hr after transfection, RBL-1 cells were treated with thapsigargin in Ca^2+^ free external solution for 5 min and then lysed in 50 mM Tris-HCl (pH 7.5), 150 mM NaCl, 1% Triton X-100, and protease inhibitors (Kar et al., 2014). Lysates were spun at 12,000 × *g* for 10 min, and the supernatant was used for immunoprecipitation reaction (anti-GFP agarose beads) at 4°C (see Supplemental Experimental Procedures for details). Bands were detected by an enhanced chemiluminescence ECL-plus western blotting detection system (GE Healthcare). Blots were analyzed by UN-Scan IT software.

### RNA Isolation and Real-Time qRT-PCR

RBL-1 cells were stimulated with thapsigargin for 5 min at room temperature in standard external solution. Thereafter, cells were washed with Ca^2+^-free external solution without thapsigargin for a further 40 min and then total RNA was extracted using an RNeasy Mini Kit (QIAGEN), as described previously (Ng et al., 2009). RNA was quantified spectrophotometrically by absorbance at 260 nm (see Supplemental Experimental Procedures for details).

### Simulations

Inter-channel spacing was estimated using a simple MatLab script, which places five points at random within a 200-nm-diameter circle, 1 million times, and then reports the mean distance (1) between any two of the points picked at random (88.5 nm) and (2) between any one point picked at random and its nearest neighboring point (47.5 nm).

Simulations of calcium diffusion within the microdomain were performed using the diffusion/heat equation with a source term for CRAC channel currents:

$$\frac{\partial C}{\partial t}=D\nabla^{2}C+I_{CRAC},$$

where C represents the concentration of calcium ions above baseline cytoplasmic levels (in μM), t is time (in μs), *D* is the diffusion constant for calcium ions (set as 300 nm^2^/μs), and $I_{CRAC}$ is the source term for influx of calcium ions through CRAC channels.

The model is set up on a 3D disc-shaped domain of the following dimensions: $r=\;\sqrt{x^{2}+y^{2}}\;\leq 100$ nm; and 0≤z≤15 nm. The top surface (*z* = 15) represents the cell membrane and the bottom surface (*z* = 0) the ER membrane; at each of these boundaries, “no flux” conditions are applied ((∂C/∂z)=0). Further specific details can be found in Supplemental Experimental Procedures.

### Statistical Analysis

Results are presented as mean ± SEM. Data were compared using Student’s t test or by ANOVA for multiple groups. Differences were considered statistically significant at values of p < 0.05.

## Supplemental Information

Document S1. Supplemental Experimental Procedures and Figures S1–S5

Document S2. Article plus Supplemental Information
