# Supplementary material for: Ca2+ Channel Re-localization to Plasma-Membrane Microdomains Strengthens Activation of Ca2+-Dependent Nuclear Gene Expression
Source: Cell Rep. 2015 Jul 2;12(2):203–16. doi: 10.1016/j.celrep.2015.06.018 (PMC4521080; doi:10.1016/j.celrep.2015.06.018)
Supplement: Document S1. Supplemental Experimental Procedures and Figures S1–S5 [file mmc1.pdf]

Cell Reports

Supplemental Information

Ca^{2+} Channel Re-localization to Plasma-Membrane Microdomains Strengthens Activation of Ca^{2+}-Dependent Nuclear Gene Expression

Krishna Samanta, Pulak Kar, Gary R. Mirams, and Anant B. Parekh

Supplemental Information

Supplemental Figure 1-related to Figure 1

Ca^{2+} entry through Orai1 channels activates c-fos and NFAT via different signalling pathways. A, The Ca^{2+} signal, following store depletion with thapsigargin, is compared between wildtype cells (WT) and cells in which Orai1 had been knocked down. B, Aggregate data from several cells are shown. Bars represent between 34 and 41 cells. In these experiments, cells were stimulated with thapsigargin in Ca^{2+} -free solution for 7 minutes before readmission of external Ca^{2+} (2 mM). C, Western blot compares Orai1 expression in wild type cells and after knockdown using an siRNA-based approach. D, Aggregate data from 3 experiments are shown. E, qPCR measurement of c-fos are compared for the different conditions. Thapsigargin (2 μM) was applied for 5 minutes. Cells were pre-treated with Synta66 (10 μM) for 10 minutes prior to stimulation. F, Images compare NFAT-dependent GFP reporter gene expression for the indicated treatments. G, Aggregate data from 4 independent experiments are compared.

Supplemental Figure 2-related to Figure 2

V102C-Orai1 induces less STAT5 phosphorylation than 100 nM thapsigargin. A, Western blot compares the extent of STAT5 phosphorlation (P-STAT5) for the conditions shown. Basal denotes resting, non-stimulated cells. Cells were stimulated with thapsigargin in Ca^{2+}-free solution for 7 minutes before 2 mM external Ca^{2+} was readmitted for 5 minutes. For cells expressing V102C-Orai1, cells were exposed to Ca^{2+}-free solution for 7 minutes before readmission of external Ca^{2+} for 5 minutes. B, Aggregate data from three experiments are compared. In these experiments, cells were cultured in the presence of La^{3+}, to reduce P-STAT5 levels in cells expressing V102C-Orai1 overnight.

Supplemental Figure 3-related to Figures 2 and 3

V102C-Orai1 locates mainly to the plasma membrane when expressed in HEK293 cells. A, Western blot compares Orai1-YFP protein levels with V102C-Orai1-YFP. B, Aggregate data from 2 independent experiments are compared. Ns denotes not significant. C, Orai1-YFP or V102C-Orai1-cherry were co-expressed. D, The spatial distribution across the cell in panel C was measured across the line scan using confocal microcopy. The green trace denotes Orai1-YFP whereas the red one denotes V102C-Orai1.

Supplemental Figure 4-related to Figure 3

Comparison of V102C-Orai1 with endogenous Orai1 on Ca^{2+} influx and c-fos expression in HEK cells. A, Ca^{2+} responses to different concentrations of thapsigargin are shown. Included are the responses to V102C-Orai1 and basal Ca^{2+} influx (perfusion in Ca^{2+}-free solution without thapsigargin followed by Ca^{2+} readmission). B, Thapsigargin dose-response curve is shown. Each point is the mean of between 17 and 26 cells. The response to V102C-Orai1 is included (red point). C, C-fos protein expression is compared between a resting cell, one

stimulated with 100 nM thapsigargin and one in which Orai1 had been knocked down 48 hours before stimulation. DAPI was used to stain the nucleus. D, Cells were transfected with STIM1 (untagged) and V102C-Orai1-cherry and C-fos expression was measured 24 hours later in a resting cell and in one stimulated with 100 nM thapsigargin. The lower panel shows an experiment in which Orai1 was knocked down and then STIM1 and V102C-Orai1-YFP expressed 24 hours later. E, Images compare c-fos expression in cells expressing V102C-Orai1-cherry in the presence of different external Ca^{2+} concentrations. F, Aggregate data from several independent experiments are compared. All cells were cultured in La^{3+}-containing medium to prevent constitutive Ca^{2+} entry through V102C-Orai1 channels from activating gene expression prior to the onset of the experiment.

Supplemental Figure 5-related to Figures 5 and 6

Expression of the either L273S-V102C-Orai1 or the SOAR domain fail to activate c-fos expression. A, Cells were transfected with L273A-V102C-Orai1 and c-fos measured under the conditions indicated. Rest denotes cells kept in Ca^{2+}-free solution before fixing, 2 Ca^{2+} represents cells exposed to Ca^{2+} free external solution for 7 minutes then 2 mM external Ca^{2+} for 5 minutes; 2 Ca^{2+} thap denotes cells exposed to Ca^{2+} free external solution for 7 minutes then 2 mM external Ca^{2+} for 5 minutes then 100 nM thapsigargin for a further 5 minutes; 2 Ca^{2+} then thap then ionom represents cells to which 2 μM ionomycin was added for 5 minutes after thapsigargin using the preceding protocol. Cells were then kept in culture medium containing La^{3+} for a further 2 hours before fixing. C-fos was measured using confocal microscopy. B, As in panel A but now the SOAR domain was expressed instead. C, Aggregate data for the conditions indicated are shown. Each bar represents > 40 cells from three independent experiments. All cells were cultured in La^{3+}-containing medium to prevent constitutive Ca^{2+} entry through V102C-Orai1 channels during the culture period.

Methods

Cell Culture and Transfection

Rat basophilic leukemia (RBL-1) and HEK293 cells were bought from ATCC and were cultured (37 °C, 5% CO2) in Dulbecco's modified Eagle medium with 10% fetal bovine serum, 2 mM L-glutamine and penicillin-streptomycin, as previously described(Kar et al., 2012). RBL-1 cells were transfected using the AMAXA system and HEK293 cells were transfected using the lipofectamine method, as described.(Kar et al., 2011)

Ca^{2+} imaging

Ca^{2+} imaging experiments were carried out at room temperature, using the IMAGO CCD camera-based system from TILL Photonics(DiCapite et al., 2009). Cells were alternately excited at 356 and 380 nm (20-msec exposures) and images were acquired every 2 seconds. Images were analysed offline using IGOR Pro for Windows. Cells were loaded with Fura 2-AM (2 μM) for 40 minutes at

room temperature in the dark and then washed three times in standard external solution of composition (in mM): NaCl 145, KCl 2.8, $CaCl_2$ 2, $MgCl_2$ 2, D-glucose 10, HEPES 10, pH 7.4 with NaOH. Cells were left for 15 minutes to allow further de-esterification. Ca^{2+}-free solution had the following composition (in mM): NaCl 145, KCl 2.8, $MgCl_2$ 2, D-glucose 10, HEPES 10, EGTA 0.1, pH 7.4 with NaOH. Low Na^+ external solution contained (in mM): NaCl 10, TRIS base 135, KCl 2.8, $CaCl_2$ 2, $MgCl_2$ 2, D-glucose 10, HEPES 10, EGTA 0.1, pH 7.4 with HCl). Ca^{2+} signals are plotted as R, which denotes the 356/380 nm ratio. Rmin was 0.40 and Rmax was 2.07.

Total internal reflection fluorescence (TiRF) microscopy

HEK293 cells expressing Orai1-YFP, V102C-Orai1-YFP or mutants thereof, were illuminated with 488-nm laser light. Light reflected from the back focal plane was detected with a x100 oil immersion objective and images were captured with 1x1 pixel binning. YFP fluorescence was measured before and then after stimulation with thapsigargin (concentrations indicated in text) for each cell. YFP fluorescence was measured in Image J along three lines drawn across each cell. To estimate the total increase in fluorescence induced by thapsigargin within the TiRF field, we integrated YFP fluorescence across the total number of pixels at rest and then after thapsigargin exposure. YFP fluorescence to thapsigargin was given as: (Total YFP fluorescence in thapsigargin-YFP fluorescence at rest)/Total YFP fluorescence in thapsigargin.

Nuclear NFAT1-GFP

NFAT1-GFP or NFAT1-cherry levels in the cytosol and nucleus was measured using the IMAGO charge-coupled device camera-based system from TILL Photonics, with a x100 oil immersion objective (numerical aperture 1.3)(Kar et al., 2012). Regions of interest of identical size were drawn in the cytosol and nucleus of each cell and calculated the nuclear/cytosolic ratio of NFAT. Only 1-3 cells per field of view on each coverslip were used and translocation was measured in these cells for up to 40 minutes.

Gene reporter assay

24–36 hours following transfection with the EGFP-based reporter plasmid that contained an NFAT promoter (gift from Dr Yuri Usachev, University of Iowa), cells were stimulated with thapsigargin and the % of cells expressing EGFP measured subsequently (~24 hours later). Gene expression was defined as fluorescence 3xSD> cell autofluorescence, measured in non-transfected cells, as described(Kar et al., 2012). Cells were stimulated in culture medium and maintained in the incubator for ~24 hours prior to detection of EGFP. In experiments were thapsigargin was the stimulus, cells were exposed to 100 nM thapsigargin for 15 minutes in culture medium before thapsigargin-containing medium was replaced with normal DMEM overnight.

siRNA knockdown

siRNAs against Orai1 and STIM1 were from Origene and siRNA against SYK was from Invitrogen, as reported previously(Ng et al., 2009).

Confocal Microscopy

After treatment, cells were fixed in 4% paraformaldehyde (PFA) at room temperature and permeabilized with PBS/Triton 0.5%. After that, cells were incubated with blocking solution (Thermo Scientific) for 1 h at room temperature. After washing with PBS/0.1% Tween20, cells were stained overnight at 4°C with c-fos primary antibody (Santa cruz Biotechnology). Fluorochrome-conjugated secondary antibody (Alexa 488, Invitrogen or Alexa 568 when YFP-tagged Orai1 constructs were expressed) for double staining was added for 1h at room temperature. Nuclei were counterstained with DAPI. Images were acquired by a Fluoview FV1000 Olympus confocal microscope with an oil immersion objective (60x 1.4 NA Plan-Apochromat; Olympus), using appropriate laser excitation.

Co-immunoprecipitation and Western blotting

48 hours after transfection, RBL-1 cells were treated with thapsigargin in Ca^{2+} free external solution for 7 minutes and then lysed in 50 mM Tris-HCl (pH 7.5), 150 mM NaCl, 1% Triton X-100, and protease inhibitors(Kar et al., 2014). Lysates were spun at 12000 × g for 10 min, and the supernatant was used for immunoprecipitation reaction (anti-GFP agarose beads) at 4°C. After washing four times with ice cold lysis buffer, lysate was resuspended in 2X SDS sample buffer. Samples were heated at 95°C for 5 min, resolved by 10% SDS–PAGE and subjected to transfer into the nitrocellulose membranes. Membranes were blocked with 5% non-fat dry milk in PBS plus 0.1% Tween 20 (PBST) buffer for 1 hour at room temperature. Membranes were washed with PBST three times and then incubated with appropriate primary antibodies for 24 hours at 4°C. Primary antibodies against total ERK 2 and Orai-1 (both Santa Cruz Biotechnology), STIM1, STAT5, Phosphorylated STAT5 and GFP (all from Cell Signaling), SYK (Abcam) were used at dilutions of 1:5000 (ERK2), 1:1000 (Orai-1, STIM1, STAT5, Phosphorylated STAT5 and GFP), 1μg/ml (SYK). The membranes were then washed with PBST again and incubated with 1:2500 dilutions of peroxidase-linked anti-rabbit (Santa Cruz Biotechnology) for 1 hour at room temperature. After washing with PBST, the bands were detected by an enhanced chemiluminescence ECL-plus Western blotting detection system (GE Healthcare). Blots were analyzed by UN-Scan IT software.

RNA isolation and real-time quantitative RT-PCR (qRT-PCR)

RBL-1 cells were stimulated with thapsigargin for 5 minutes at room temperature in standard external solution. Thereafter, cells were washed with Ca^{2+}-free external solution without thapsigargin for a further 40 minutes (at room temperature) and then total RNA was extracted using an RNeasy Mini Kit (Qiagen), as described previously(Ng et al., 2009). RNA was quantified spectrophotometrically by absorbance at 260 nm. Total RNA (1 μg) was reverse-transcribed using the iScriptTM cDNA Synthesis Kit (Bio-Rad), according to the manufacturer's instructions. To quantify mRNA levels, we performed real-time

PCR by using an ABI7000 instrument (Applied Biosystems) and then detected the fluorescence of samples in 96-well plates by using Taq Man Gene Expression Assays (Applied Biosystems), according to the manufacturer's instructions. Each 10 µl PCR reaction contained the cDNA, H_2O, the Master Mix (Applied Biosystems) and Probe & Primer Mix (Applied Biosystems). The mRNA levels were normalised to β-actin. Data were analysed using ABI7000 System Software.

Statistical Analysis

Results were presented as mean±sem. Data were compared using Student's t test or by analysis of variance (ANOVA) for multiple groups. Differences were considered statistically significant at values of $p<0.05$.

DiCapite, J.L., Shirley, A., Nelson, C., Bates, G., and Parekh, A.B. (2009). Intercellular calcium wave propagation involving positive feedback between CRAC channels and cysteinyl leukotrienes. FASEB Journal *23*, 894-905.

Kar, P., Nelson, C., and Parekh, A.B. (2011). Selective activation of the transcription factor NFAT1 by calcium microdomains near Ca2+ release-activated Ca2+ (CRAC) channels. Journal of Biological Chemistry *286*, 14795-14803.

Kar, P., Nelson, C., and Parekh, A.B. (2012). CRAC channels drive digital activation and provide analog control and synergy to Ca2+-dependent gene regulation. Current Biology *22*, 242-247.

Kar, P., Samanta, K., Kramer, H., Morris, O., Bakowski, D., and Parekh, A.B. (2014). Dynamic assembly of a membrane signaling complex enables selective activation of NFAT by orai1.

. Current Biology *24*, 1361-1368.

Ng, S.-W., Nelson, C., and Parekh, A.B. (2009). Coupling of Ca2+ microdomains to spatially and temporally distinct cellular responses by the tyrosine kinase Syk. Journal of Biological Chemistry *284*, 24767-24772.

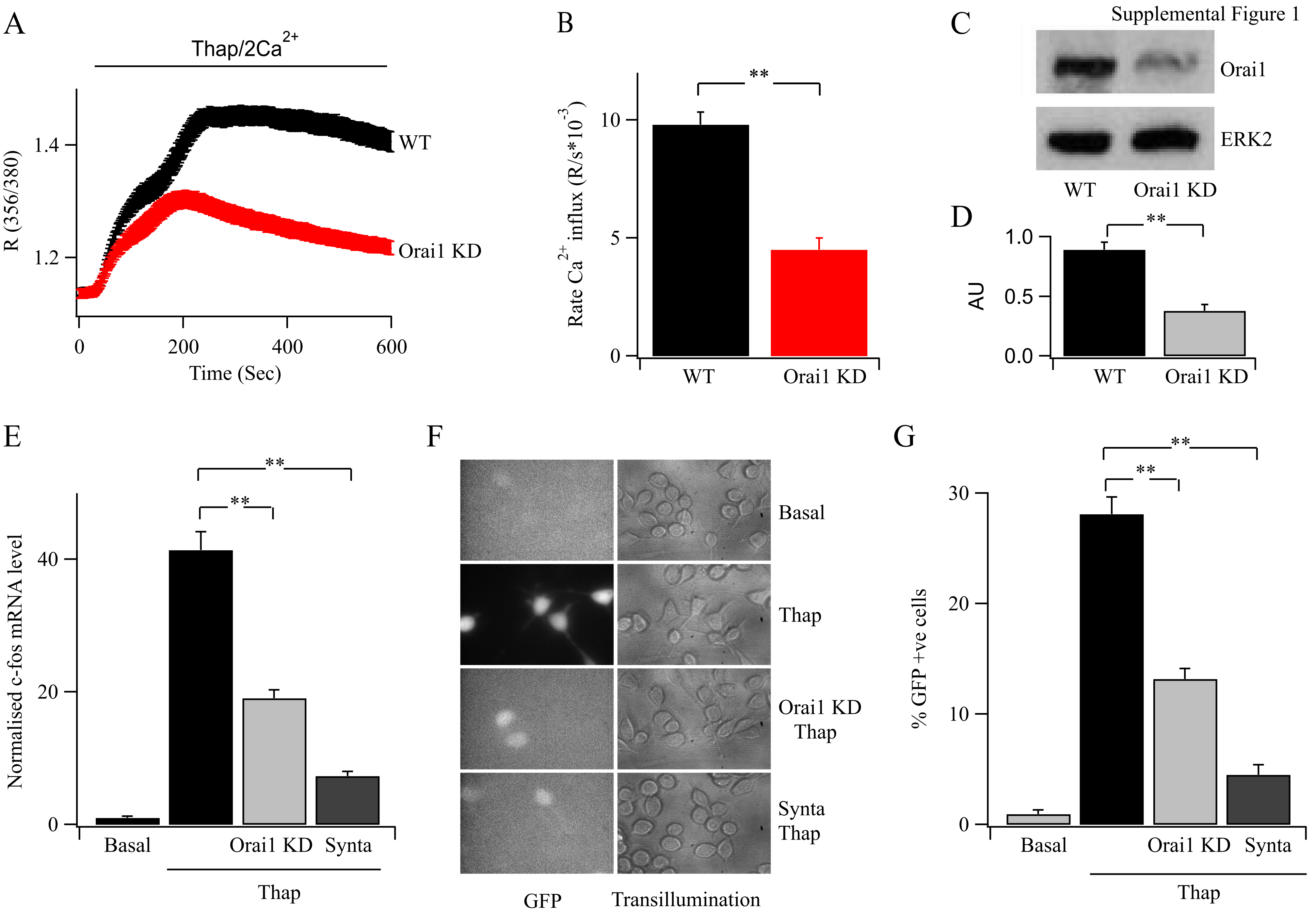
Supplemental Figure 1
A
Thap/2Ca2+
R (356/380)
1.4
1.2
WT
Orai1 KD
0
200
400
600
Time (Sec)
B
Rate Ca2+ influx (R/s*10-3)
10
5
0
**
WT
Orai1 KD
C
Orai1
ERK2
WT
Orai1 KD
D
AU
1.0
0.5
0.0
**
WT
Orai1 KD
E
Normalised c-fos mRNA level
40
20
0
**
**
Basal
Orai1 KD
Synta
Thap
F
Basal
Thap
Orai1 KD Thap
Synta Thap
GFP
Transillumination
G
% GFP +ve cells
30
20
10
0
**
**
Basal
Orai1 KD
Synta
Thap

Supplemental Figure 2

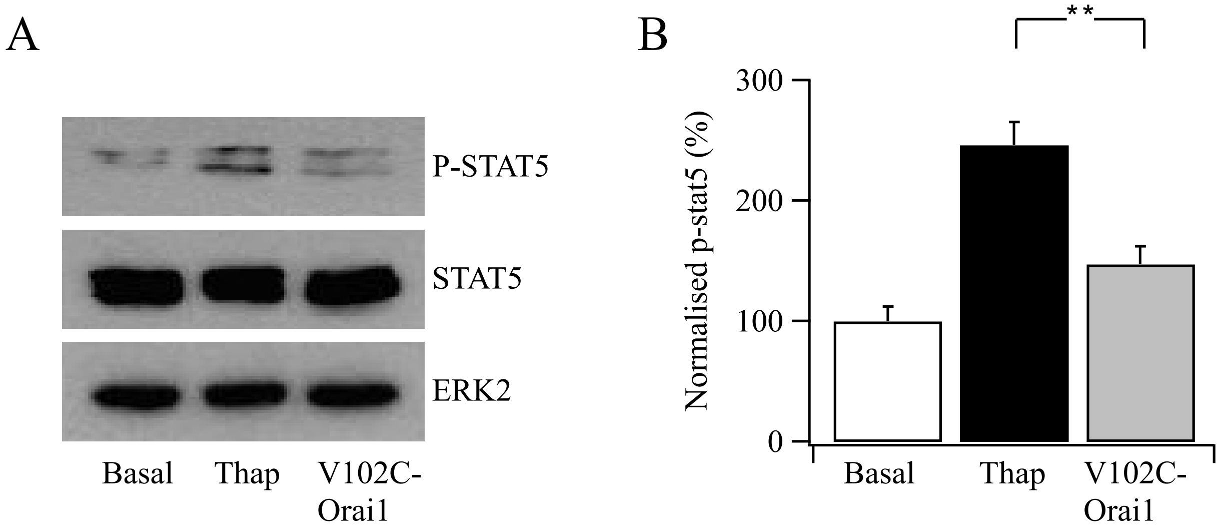

Supplemental Figure 3

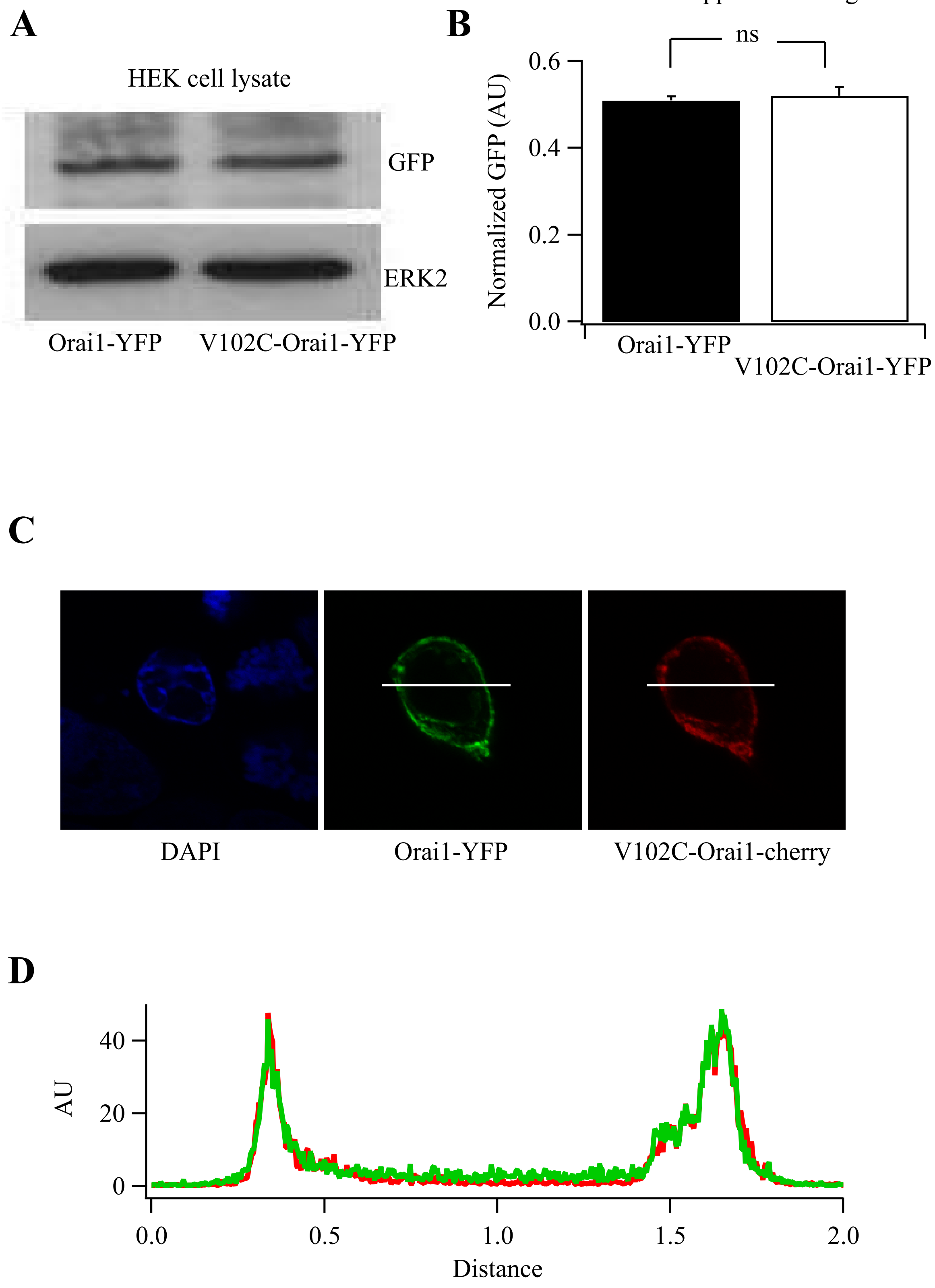

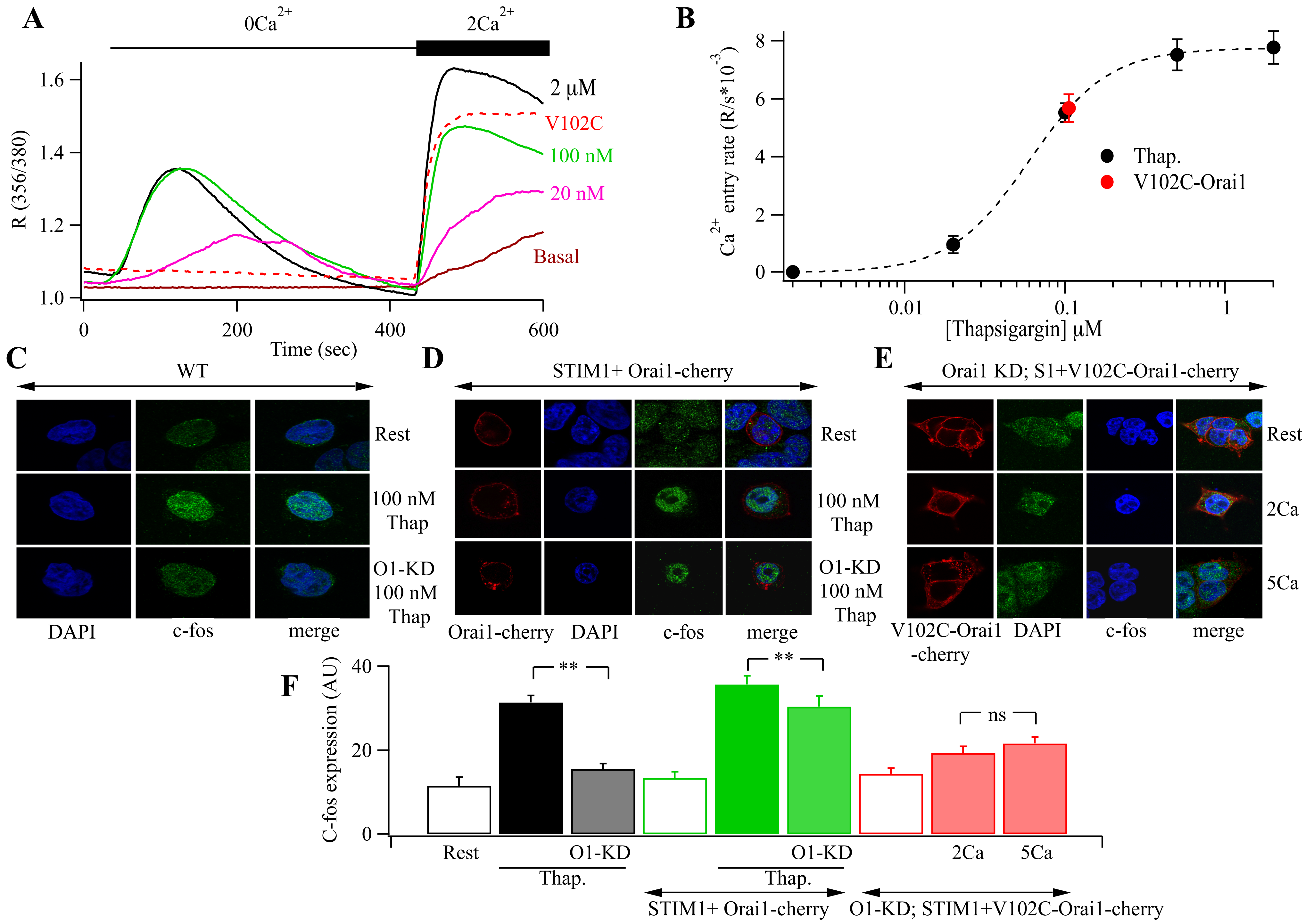
A
0Ca2+
2Ca2+
2 μM
V102C
100 nM
20 nM
Basal
R (356/380)
Time (sec)
B
Ca2+ entry rate (R/s*10-3)
Thap.
V102C-Orai1
[Thapsigargin] μM
C
WT
Rest
100 nM Thap
O1-KD 100 nM Thap
DAPI
c-fos
merge
D
STIM1+ Orai1-cherry
Orai1-cherry
E
Orai1 KD; S1+V102C-Orai1-cherry
2Ca
5Ca
V102C-Orai1 -cherry
F
C-fos expression (AU)
**
ns
Rest
O1-KD
Thap.
2Ca
5Ca
STIM1+ Orai1-cherry
O1-KD; STIM1+V102C-Orai1-cherry

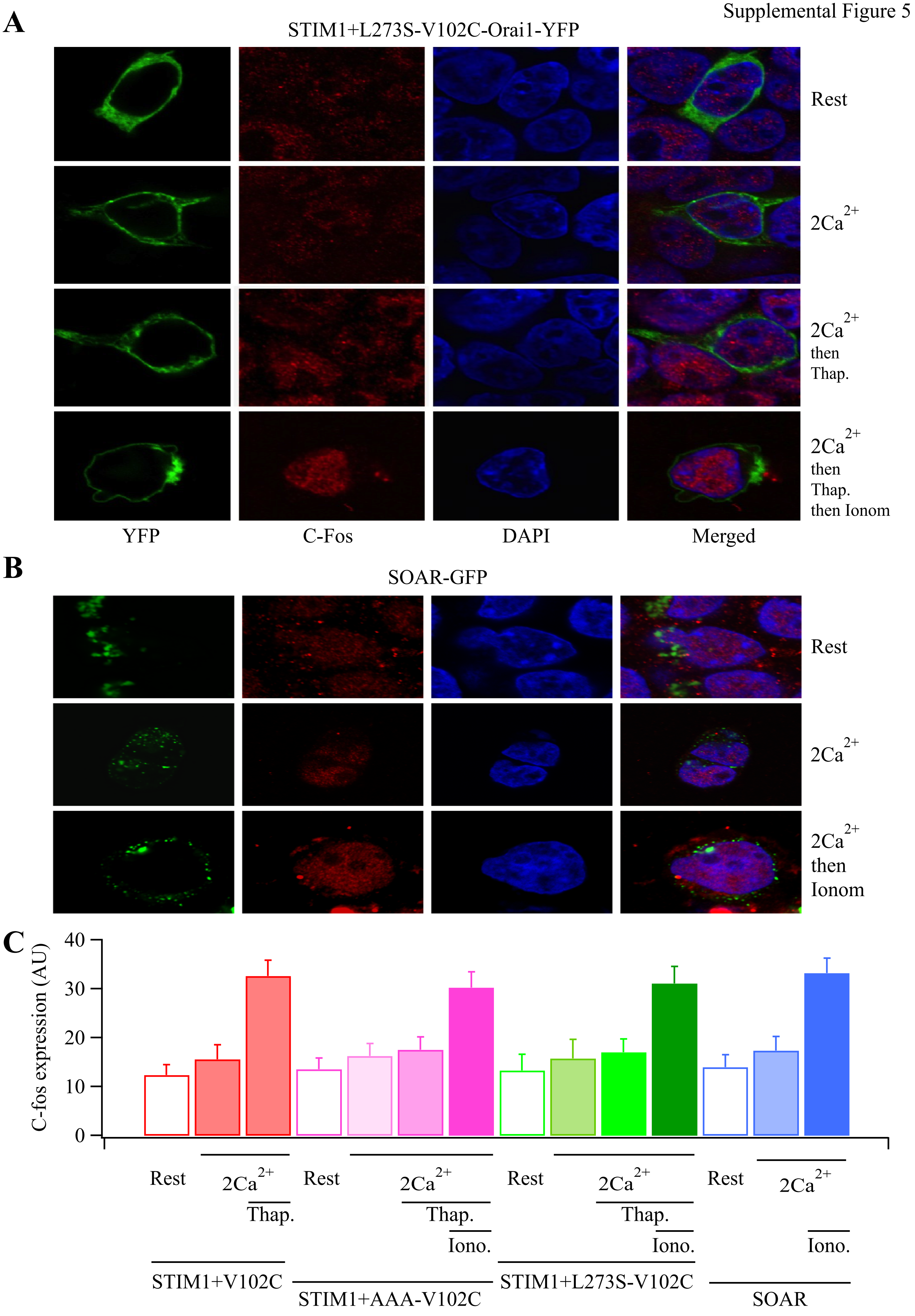
A
STIM1+L273S-V102C-Orai1-YFP
Rest
2Ca2+
2Ca2+ then Thap.
2Ca2+ then Thap. then Ionom
YFP
C-Fos
DAPI
Merged
B
SOAR-GFP
Rest
2Ca2+
2Ca2+ then Ionom
C
C-fos expression (AU)
0
10
20
30
40
Rest
2Ca2+
Thap.
Iono.
STIM1+V102C
STIM1+AAA-V102C
STIM1+L273S-V102C
SOAR
